# Bioluminescence Imaging-Based Assessment of the Anti-Triple-Negative Breast Cancer and NF-Kappa B Pathway Inhibition Activity of Britanin

**DOI:** 10.3389/fphar.2020.00575

**Published:** 2020-05-05

**Authors:** Xinyi Xu, Yingying Guo, Getao Du, Huifang Liu, Lin Wang, Dan Chen

**Affiliations:** ^1^Engineering Research Center of Molecular and Neuroimaging, Ministry of Education, School of Life Science and Technology, Xidian University, Xi'an, China; ^2^School of Information Sciences and Technology, Northwest University, Xi'an, China

**Keywords:** bioluminescence imaging, Britanin, triple-negative breast cancer, phytochemical, nuclear factor-kappa B pathway

## Abstract

Britanin has been reported to have therapeutic effects on neurodegenerative and inflammation-based diseases. However, whether it is involved in the regulation of triple-negative breast cancer development has not been elucidated. In this study, we investigated the anti-tumor activity against triple-negative breast cancer tumor of Britanin by bioluminescence imaging *in vivo* using athymic (nu/nu) mice implanted with MDA-MB-231 and SUM-159 cells expressing a luciferase reporter gene, and explored the anti-tumor mechanism of Britanin. The results showed that Britanin treatment inhibited triple-negative breast cancer cell proliferation *in vivo*, and Cell Counting Kit-8 (IC_50_ values are 4.27 and 5.05 μM) and colony formation tests (P < 0.001) confirmed this result. Transwell assays were conducted to verify that Britanin treatment inhibited cell migration and invasion (P < 0.001). Apoptosis was determined by TdT-mediated dUTP nick-end labeling method. Western blot and qRT-PCR analysis showed that Britanin treatment caused a decrease in the member expression of NF-kappa B signaling pathway. Computational modeling showed that Britanin could directly bind to a p-65 core region composed of Cys38, Cys120, and Gln128 residues. The results showed that the inhibitory mechanisms of Britanin on cancer cells may be by ways of inhibiting the NF-kappa B pathway. In addition, bioluminescence imaging screening system is useful for accelerating the application of Britanin in the antitumor field, and provides a useful tool for evaluating the phytochemicals efficacy in inhibiting cancer cell proliferation in animal models.

## Introduction

In 2018 approximately 2.1 million newly diagnosed female breast cancer cases accounted for almost one in four cancer cases among women (Bray et al., 2018). There are many therapeutic approaches to treat breast cancer including chemotherapy to inhibit the growth of cancer cells. Most of the current chemotherapeutic agents, including anthracyclines, hormone drugs, aromatase inhibitors, and biological drugs, are antimetabolites or target specific hormone receptors (Carels et al., 2016). Researchers have found that sequential single-drug chemotherapy has an indefinite effect on progression-free survival, while combined chemotherapy has an increased the efficacy of chemotherapy (Dear et al., 2013). Experiments results proved that the application of natural product-based agents in combination with chemotherapeutic drugs is a productive approach to the treatment of breast cancer (Israel et al., 2018). Phytochemicals, such as isoflavones (Shao et al., 1998), epigallocatechin gallate (Sen and Chatterjee, 2011), resveratrol (Gomez et al., 2013), curcumin (Cheng et al., 2001), lignans (Saarinen et al., 2007), and carotenoids (Dorjgochoo et al., 2009), have proven effective at inhibiting tumorigenesis, suppressing breast cancer cell growth, and even increasing the apoptosis of breast cancer cells. The above experiments have shown that combining chemotherapeutic agents with phytochemicals is a plausible novel strategy for treating breast cancer patients. Some works suggest that in 50% of tumors, the nuclear factor-kappa B (NF-κB) pathway is constitutively activated in a variety of cancer types, including breast cancer. It is very meaningful to develop a new anti-cancer drug for the NF-κB pathway. Britanin is a guaiacyl-type sesquiterpene lactone extracted from *Inula britannica*. Previous studies have shown that Britanin inhibits the NF-κB pathway by inhibiting the degradation of IκB-α, nuclear translocation of NF-κB, and NF-κB/DNA binding activity (Park et al., 2014).

The pharmacological activity of phytochemicals has mainly been studied by cell-level methods or histopathological methods *in vitro*, but the traditional methods mentioned above still have some limitations. First, the amount of animal samples that the experiment needed is huge, the statistical results of the data are complicated and the workload is great (Johnson et al., 2001). Second, histopathological sections provide quantitative information on the section, and the measurement of the volume of the tumor cannot achieve the accuracy of three-dimensional analyses (Dear et al., 2013). Third, as traditional methods are invasive, which cannot allow for continuous studies in a single animal and cannot provide important information on the optimal timing and dosing of drugs (Rudin and Weissleder, 2003). In order to find more effective tumor inhibition natural phytochemicals, a range of imaging techniques for discovering new drug have been used. Optical imaging techniques, such as fluorescence imaging and bioluminescence imaging (BLI), are inexpensive, which have high-throughput capabilities, do not require the steps of radionuclides labeling and enable semiquantitative analyses *via* the measurement of fluorescence intensity per unit area, have been used to monitor tumor tissue growth and metastasis. It is also used to monitor the release and diffusion of trace drugs *in vivo*. Jenkins et al. (2003) injected human prostate cancer cells expressing the luciferase gene into mice and used an *in vivo* bio-optical imaging system to monitor the recurrence and metastasis of prostate cancer cells after chemotherapy in real-time and *in vivo*. Due to the subcellular resolution of optical imaging systems, cellular heterogeneity within organs can be quantified and monitored in drug screens (Walsh et al., 2014). These studies have identified that optical imaging techniques have the potential to increase the efficiency of drug screening, assess the pharmacokinetics of new drugs, and evaluate drug effects *in vivo*.

In this paper, the BLI method was used to assess whether Britanin has anti-breast cancer activity *in vivo*. To investigate the mechanism of the anticancer action of this compound, CCK-8 method and colony forming test were used to evaluate the effect of Britanin on inhibiting tumor cell proliferation. The effects of Britanin on cell migration and invasion were estimated by Transwell assays. The effect of Britanin on tumor cell apoptosis was evaluated by the TdT-mediated dUTP nick-end labeling (TUNEL) method. And the western blot and qRT-PCR method were performed to detect the expression of the proteins of related signaling pathways. A molecular docking simulation was performed to identify whether Britanin could bind to the NF-κB pathway protein p-65 *via* possible covalent binding sites.

## Materials and Methods

### Cell Lines and Reagents

Human breast cancer cells included MDA-MB-231 cells, MDA-MB-231 luc cells, SUM-159 cells, and SUM-159 luc cells (provided by Xi'an Medical University) were incubated at 37°C with 5% CO_2_ in RPMI-1640 (GIBCO) supplemented with 10% fetal bovine serum (FBS, HyClone, Thermo Scientific), penicillin (100 IU/ml), and streptomycin (100 mg/ml). Cells were passaged three times a week. The Britanin working solutions (10 mM Britanin dissolved in DMSO) provided by Shanghai Jiaotong University were prepared by dilution of the stock solution in fresh culture medium on the day of use. Britanin, as a natural product, was purified by high-performance liquid chromatography and characterized by nuclear magnetic resonance (NMR) spectroscopy. The purity of Britanin was greater than 95% (**Figure S1**). A Cell Counting Kit-8 (Dojindo), Hoechst staining kit (Beyotime), TUNEL Apoptosis Detection kit (Beyotime), D-Luciferin potassium salt (Sciencelight), and antibodies (Abcam) were used in this study. Total RNA RNA extraction and CDNA synthesis used RNAiso Plus and the Reverse Transcription System (TaKaRa, Tokyo, Japan). Quantitative RT-PCR (qRT-PCR) analysis was performed in a 7300 Real-Time System (ABI, New York, America) using the SYBR Green RealMasterMix (TIANGEN, Beijing, China).

### Cell Viability Assay

Measurements of cell viability at different drug concentrations were performed. MDA-MB-231 cells, MDA-MB-231 luc cells, SUM-159 cells, and SUM-159 luc cells (1×10^4^ cells/ml) were seeded in 96-well plates (100 μl) at 37°C overnight with 5% CO_2_ and then incubated with various concentrations of Britanin (0.33, 1, 3, 9, 27, and 81 μM) at 37°C for 72 h. Four wells containing only complete medium were used as blank control group, and four wells containing tumor cells suspended in the complete medium which were used as control group. Thiazolyl blue tetrazolium bromide (0.5 mg/ml, 10 μl) was added to each well. After incubation for 4 h, using a Multiskan Ascent microplate photometer, the absorbance was measured at 492 nm wavelength. The control group cells were regarded as having a 100% survival rate. And the percentage of growth inhibition was calculated as cell growth inhibition (%) = (treated OD-blank OD)/(control OD-blank OD) × 100%. The concentration required for a 50% inhibition of viability (IC_50_) was then determined.

### Colony Formation Assay

MDA-MB-231 luc and SUM-159 luc cells (500 cells/well) were seeded in 12-well flat-bottomed plates and incubated for 24 h. Using the complete medium as the control group and different concentrations of Britanin as the experimental group, MDA-MB-231 luc cells and SUM-159 luc cells were tested. Cells were pretreated with different concentrations (2, 4, or 8 μM) of Britanin for 48 h. The treatment medium was replaced with normal growth medium and was replaced with normal growth medium every 3 d; After 2 weeks of incubation, formed colonies were fixed with 4% paraformaldehyde for 10 min, stained with 0.5% crystal violet for 10 min, washed again with PBS and photographed. The results were quantified with Adobe Photoshop Software.

### Transwell Migration Assay

For the Transwell migration experiment, cells were seeded into the upper Matrigel filled chamber of a 24-well Transwell plate with 200 μl of serum-free medium. The MDA-MB-231 and SUM-159 cells density were adjusted to 2×10^5^ cells/ml. Then, Britanin with different concentrations (2, 4, 6 μM) were added to the cells. Four wells containing the solution served as the control group. Simultaneously, 500 μl of 10% FBS-supplemented medium was added to the lower chamber. Cells were removed from the upper Matrigel chamber membrane using a cotton swab after 24 h of incubation and were attached to a glass slide, fixed with methanol and stained with Giemsa. Three fields per chamber were analyzed by light microscopy for migrated cell quantification. The experiment was repeated three times.

### Animal Studies

All animal studies carried out in compliance with the Guide for the Care and Use of Laboratory Animal Resources and approved by the University of Xi'an Jiaotong Animal Care and Use Committee (number XY-AUC-2017-213). Two weeks after the implantation of cancer cells (5×10^6^ cells/site), Britanin was intraperitoneally injected. Model mice were randomly divided into two or three groups (control group [0 mg/kg] and test group [5 mg/kg], or high dose group [10 mg/kg], low dose group [5 mg/kg] and control group [0 mg/kg]). Britanin dissolved in PBS. Mice were treated once every other day. The size of the tumor *in vivo* and the weight of mice were measured and compared with that of the xenograft tumors in mice of the placebo control. BLI was recorded during the experiment. D-luciferin (30 mg/ml^−1^, 100 μl) was injected with the mice 10 min before BLI. The animals were then anesthetized with 2% isoflurane and 0.3 L/min of oxygen. The area of regions of interest (ROIs) image processing and analysis visualized using Live Image 4.5 software. To quantitative analysis, the ROIs of the tumor were evaluated with a white light image, and muscle regions of similar size (opposite positioning with the tumor) were selected as muscle ROI. The background value is subtracted from each of the luminescence images. The average of the fluorescence signal within each ROI is then calculated. Simultaneously, tumor volume was also calculated *in vitro* using calipers. After the mice were sacrificed, tumor volume was calculated with calipers, tumors and organs were fixed with 10% formalin solution.

### Histological Analysis

The *in vivo* toxicity of Britanin was analyzed through H&E histological staining of the major organs. The tumors, liver, hearts and kidneys were embedded in tissue freezing medium, frozen to −80°C and then cut into slices with a thickness of approximately 8 µm. These cell tissue slices were then stained using DAPI and imaged using confocal laser scanning microscopy.

### TdT-Mediated dUTP Nick End Labeling Assay

The paraffin-embedded tumor tissue sections were treated with fresh xylene and a gradient ethanol solution several times for dewaxing and rehydration at room temperature. The slides were incubated with protease K (20 μg/ml) in PBS for 30 min and then immersed in 4% paraformaldehyde for 5 min at room temperature. The slides were rinsed twice with PBS for 5 min per rinse. Then, the slides were equilibrated with 100 μl of equilibration buffer at room temperature for 5 min, and the slides were incubated with 100 μl of rTdT reaction mix per slide at 37°C in a humidified chamber for 1 h. Next, the slides were immersed in 2×SSC at room temperature for 15 min. Each slide was incubated with 100 μl of streptavidin HRP solution for 30 min and washed twice in PBS for 5 min per wash at room temperature. The slides were washed twice again with PBS for 5 min per wash at room temperature. At the last step, diaminobenzidine peroxidase substrate was added, and the slides were analyzed by light microscopy. The excised tumors were also stained with hematoxylin-eosin (H&E).

### Western Blot and Quantitative RT-PCR Analysis

The cells were cultured at 0 and 5 mg Britanin for 24 h respectively. The cells were subsequently lysed using buffer with protease inhibitors. Total protein (30 µg) were separated by sodium dodecyl sulfate-polyacrylamide gel electrophoresis (10%) and transferred to polyvinylidenedifluoride membranes. Membranes were blocked with 5% fat-free milk/TBST and incubated with primary antibodies and secondary antibodies. Antibodies against p-65, p150/p50, p-p65, and housekeeping gene, GAPDH were obtained from Abcam. Secondary antibodies were HRP-conjugated antibodies and visualized by a chemiluminescent reagent (The original data of Western Blot in **Data Sheet 1**).

The cells were cultured in 0 or 5 mg Britanin for 24 h respectively, homogenized and suspended with buffer for the RNA isolation and cDNA synthesis according to the manufacturer's instruction. The PCR parameters were same as manufacturer's instruction. Oligonucleotide primers for p-65, p-50, and GAPDH were as follows: oligonucleotide sequence of p-50 (217 bp), p50F: 5′-AGTAGCTGAGAGGCACATGG-3′, p50R: 5′-AGCGCACTCCAACCTTCTCA-3′, oligonucleotide sequence of p65 (256 bp), p65F: 5′-GCACTTACGGATTCTGGTGG-3′, p65R: 5′-GCACTTACGGATTCTGGTGG-3′, oligonucleotide sequence of GAPDH (252 bp), GAPDHF: 5′-CACTGGCATGGCCTTCCGTG-3′, GAPDHR: 5′-GAAATGAGCTTGACAAAGTG-3′. The quantification of each sample of cDNA was performed in triplicate, each PCR was replicated three times for verification, and the 2^−ΔΔCT^ method was used to analyze the relative changes in gene expression from the qPCR experiments.

### Molecular Docking Simulation Method

In this study, the monomer in the p-65 was used as the initial structure and receptor protein (PDB coded: 1IKN) (Huxford et al., 1998). Cys38 of p-65 was set as the center of the docking box, and the size of the box was set to 40 Å × 40 Å ×40 Å. Using the Tripos standard force field and Powell energy gradient method, the low energy stable conformation of the small molecule was obtained after 100 optimization iterations. All docking simulations were performed using AutoDock Vina software (Sanner, 1999).

### Statistical Analysis

Data are presented as the mean values ± SD of independently repeated experiments, with values of P < 0.05 considered to represent a statistically significant difference between compared data.

## Results

### Cell Viability Assay

As indicated in **Figure S2**, Britanin had apparent antigrowth activity in the four cell lines in a concentration-dependent manner. At a 9 μM Britanin treatment concentration, the cell survival rates of MDA-MB-231 luc cells, MDA-MB-231 cells, SUM 159 luc cells, and SUM 159 cells were approximately 8.9%, 9.0%, 16.4%, and 22.0% after 72 h. The half-maximal inhibitory concentrations (IC_50_) of Britanin in MDA-MB-231 luc, MDA-MB-231, SUM 159 luc, and SUM 159 cell lines were 4.27, 4.41, 5.05, and 5.33 μM, respectively. Luc-labeled cells and non-Luc-labeled cells showed the same reactions to Britanin, so Luc-labeled cells were used in subsequent experiments.

### The Antiproliferation Effect of Britanin

**Figure 1:**
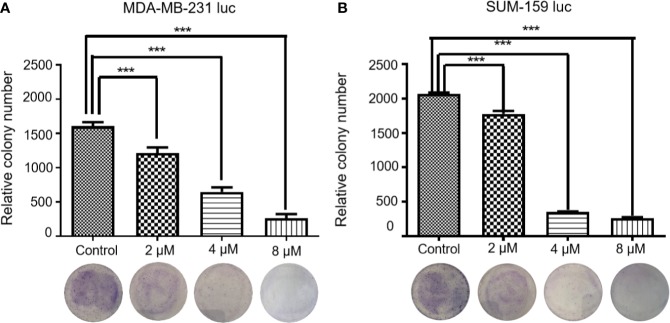
Representative images of MDA-MB-231 luc cells **(A)** and SUM-159 luc cells **(B)** treated with Britanin (2, 4, and 8 μM) or complete medium as the control group for 48 h and were stained with crystal violet. Data are expressed as the mean ± S.D. of three independent experiments. ***P < 0.001 vs. untreated cells.

As shown in **Figure 1**, Britanin treatment significantly inhibited colony formation in two cell lines. The number of MDA-MB-231 luc and SUM-159 luc cell colonies decreased by more than 50% when cells were pretreated with 4 μM Britanin. Britanin pretreatment at 8 μM eliminated more than 90% of the colony formation in MDA-MB-231 luc cells and SUM-159 luc cells. The differences in numbers of cell colonies in the two groups were significant. Therefore, the results of the above test suggest that Britanin inhibits breast cancer cell proliferation in a dose-dependent manner *in vitro*.

### Effect of Britanin on Migration and Invasion in Transwell Assays

**Figure 2:**
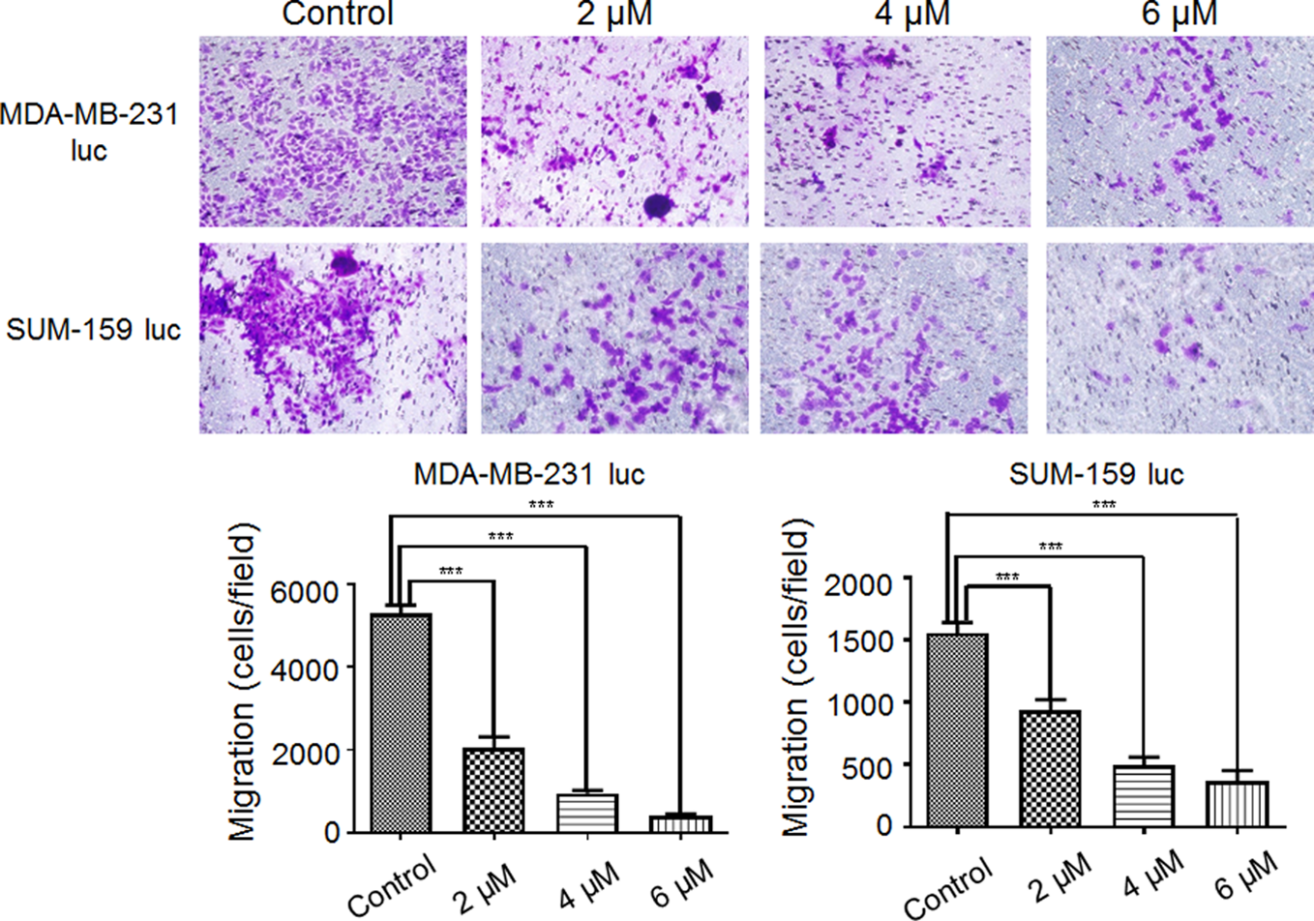
Effect of Britanin on the migration of MDA-MB-231 luc cells and SUM-159 luc cells. Transwell chambers were used to detect the ability of cells to migrate (×100, magnification). Cells were treated with 0, 2, 4, or 6 μM Britanin for 24 h. The percent cell migration is shown. The error bars represent three independent experiments and each experiment was repeated three times. ***P < 0.001 vs. untreated cells.

As shown in **Figure 2**, the data revealed that Britanin treatment notably restrained MDA-MB-231 luc cell (48% inhibition at 2 μM, 83% inhibition at 4 μM) migration and markedly abated SUM-159 luc cell migration and invasion (41% inhibition at 2 μM, 80% inhibition at 4 μM). The differences in numbers of cell in the test group and control group were significant. Therefore, these data support the hypothesis that Britanin inhibits the migration of breast cancer cells.

### Britanin-Mediated Reduction of Tumor Growth *In Vivo*

**Figure 3:**
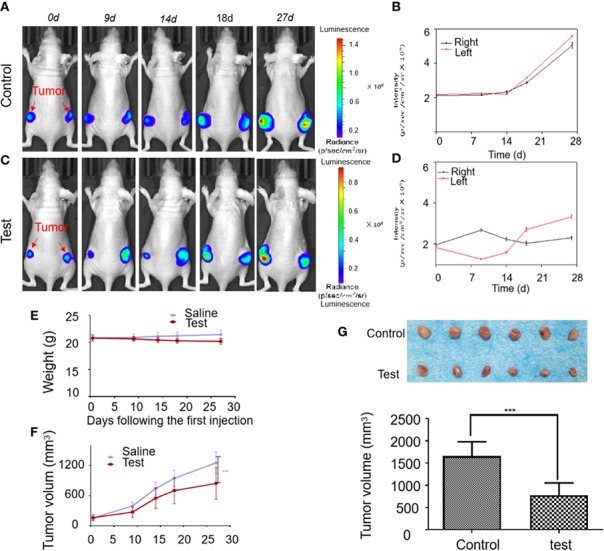
The activity evaluation of Britanin on breast cancer model mouse *in vivo*. **(A)** Representative bioluminescence images in an untreated control group of MDA-MB-231 luc inoculated mice tumor model. **(B)** Quantification of bioluminescence intensity by ROIs that encompass the tumor. Data represent as the means ± S.D. **(C)** Representative bioluminescence images recorded before and after injection Britanin of 5 mg/kg every 2 d for 27 d by intraperitoneal injection in MDA-MB-231 luc inoculated mice tumor models as the test group. **(D)** Quantification of the tumor bioluminescence ROIs. Data represent as the means ± S.D. **(E)** The mouse was weighed before and after Britanin injections, and there was no significant difference with an untreated control group. **(F)** Tumor volume was calculated before and after Britanin injections. **(G)** Photograph of excised tumors from the control, test treatment group. The graph represents the average volume of the tumor. ***P < 0.001.

The tumor tissues growth inhibition activity of Britanin on MDA-MB-231 luc cells transplanted in mice (n=6, two groups) was studied by BLI continuously *in vivo*. Initially, 1×10^5^ cells were implanted into the bilateral legs of mice. At 2 weeks post-implantation, when the luminescence signal of cells reached 2×10^5^ p/s/cm^2^/sr, the mice were injected with Britanin once every 2 d or the placebo. By the 14th day post-treatment, the growth of the luminescence signal of MDA-MB-231 luc cells implanted in mice treated with Britanin (the average was 2.02×10^5^ p/s/cm^2^/sr, **Figure 3C**) was lower than that in mice of the placebo control group (2.27 ×10^5^ p/s/cm^2^/sr, **Figure 3A**). By the 27th day post-treatment, the growth of the luminescence signal of MDA-MB-231 luc cells implanted in mice treated with Britanin (2.81×10^5^ p/s/cm^2^/sr, **Figure 3D**) was significantly lower than that in mice of the placebo control group (5.31 ×10^5^ p/s/cm^2^/sr, **Figure 3B**). The mice in the Britanin treatment group lost an average of 5.8% in weight during the treatment (**Figure 3E**). Another experiment was carried out at the same time. On the 27 day post-treatment, using the calipers to calculate with the tumor volume, the average volume of the MDA-MB-231 luc xenograft tumors in athymic nu/nu mice treated with Britanin (843.2 ± 34.2 mm^3^) was smaller than that of MDA-MB-231 luc tumors in the mice of the placebo control (1253.6 ± 48.0 mm^3^, **Figure 3F**). The differences in volume of tumors in the test group and control group were significant. The resulting showed that an average increasing in tumor volume of test group approximately 5.2 times and an average increasing in tumor volume of test group approximately 8.0 times, relative to the baseline volume. At the end of the 27th day, the excised tumors were weighed after euthanasia, and the average tumor volume of the Britanin-treated group was decreased by 53% compared to that of the control group (**Figure 3G**). By the measurement of the micrometer caliper *in vitro*, the differences in volume of tumors in the test group and control group were significant.

We performed the same experiments with a mouse xenotransplant model of SUM-159 luc cells. The results showed that the *in vivo* bioluminescence signal of the test group tumor was significantly lower than that of the placebo control group and suggested that the growth rate of tumors was inhibited by Britanin (**Figure S3A**). The daily bioluminescence signals were plotted to show that the bioluminescence signals in the high-dose group increased slowly over time, while those in the placebo control group showed an increased growth trend from 13th day (**Figure S3B**). The mice in the Britanin treatment group gained weight normally and showed no signs of discomfort during the treatment (**Figure S3E**). The tumors in athymic nu/nu mice treated with Britanin were significantly smaller than those in the mice of the placebo control group (P < 0.001, **Figures S3D–F**).

### Histology

**Figure 4:**
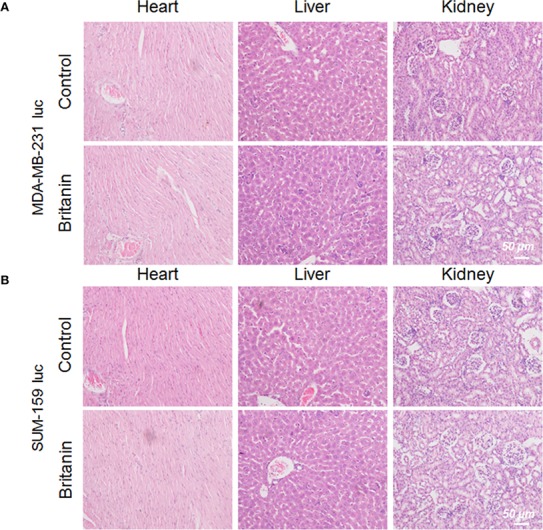
Histological analysis of organs from mice in the control group and Britanin treatment group. **(A)** Histological analysis of major organs (including heart, liver and kidneys) from mice xenotransplant model of MDA-MB-231 luc cells in the control group (with saline as control) and Britanin treatment (5 mg/kg) group. **(B)** Histological analysis of major organs (including heart, liver and kidneys) from mice xenotransplant model of SUM-159 luc cells in the control group (with saline as control) and Britanin treatment (5 mg/kg) group.

After 27 d of continuous treatment, involving the intraperitoneal injection of Britanin (5 mg/kg) into BALB/c mice xenotransplant model of SUM-159 luc cells or MDA-MB-231 luc cells, the major organs (heart, liver, and kidneys) were harvested for histopathological analysis. No noticeable tissue or cellular damage was observed in the H&E-stained organ slices from Britanin-treated mice compared to those from nude mice treated with saline as control, except for some binuclear nucleolar cells in the liver (**Figure 4**).

### TUNEL Assay, Immunohistochemical, and qRT-PCR Results

**Figure 5:**
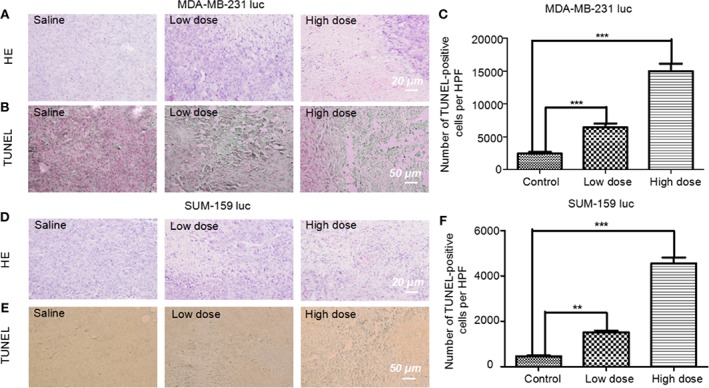
Pathological analysis of tumor tissue treated with or without Britanin using H&E staining and apoptosis assay with the TUNEL stain. **(A)** Histological analysis of tumor tissue from mice xenotransplant model of MDA-MB-231 luc cells in the control group, low dose (5 mg/kg), and high dose (10 mg/kg) Britanin treatment group. **(B)** TUNEL assay on the tissue-sample sections from mice xenotransplant model of MDA-MB-231 luc cells, where the apoptotic cells are stained brown. **(C)** TUNEL assay on the tissue-sample from mice xenotransplant model of SUM-159 luc cells in the control group, low dose (5 mg/kg), and high dose (10 mg/kg) Britanin treatment group. The graph represents the number of apoptotic cells on a high-power field (n = 6), data represent as the means ± S.D., ***P < 0.001. **(D)** Histological analysis of tumor tissue from mice xenotransplant model of SUM-159 luc cells in the control group, low dose (5 mg/kg), and high dose (10 mg/kg) Britanin treatment group. **(E)** TUNEL assay on the tissue-sample sections from mice xenotransplant model of SUM-159 luc cells, where the apoptotic cells are stained brown. **(F)** TUNEL assay on the tissue-sample from mice xenotransplant model of SUM-159 luc cells in the control group, low dose (5 mg/kg), and high dose (10 mg/kg) Britanin treatment group. The graph represents the number of apoptotic cells on a high-power field (n = 6), data represent as the means ± S.D., **p < 0.01, ***p < 0.001.

**Figure 6:**
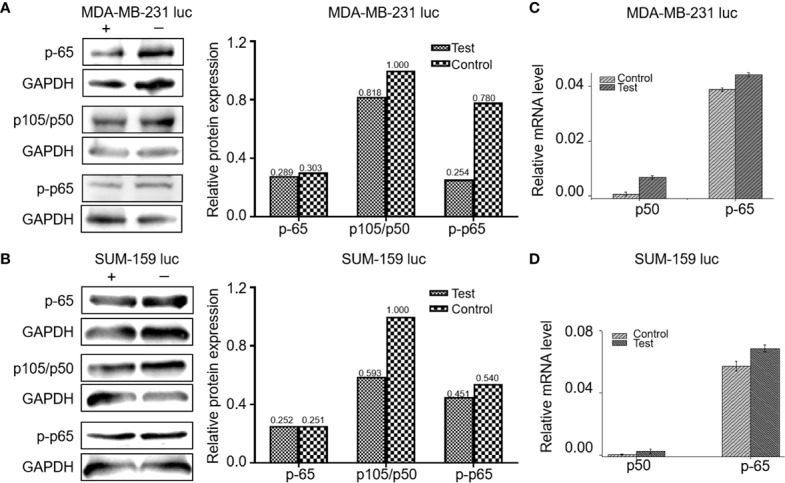
Britanin regulates the proteins levels of NF-κB p-65, p105/50, p-p-65, and regulates the mRNA levels of NF-κB p-65, p105/50 from cells treated with or without Britanin for 24 h. **(A)** Western blots of proteins extracted from MDA-MB-231 luc cells treated with or without Britanin with the indicated antibodies: NF-κB p-65, p105/50 and p-p-65, GAPDH was used as a control. The data of relative protein expression of NF-κB pathway presented in bar charts. **(B)** Western blots of proteins extracted from SUM-159 luc cells treated with or without Britanin with the indicated antibodies: NF-κB p-65, p105/50, p-p-65, GAPDH was used as a control. The data of relative protein expression of NF-κB pathway presented in bar charts. **(C)** Relative mRNA concentrations of NF-κB p-65, p105/50 in MDA-MB-231 luc cells. Data represent as the means ± S.D. of three independent experiments (n=3). **(D)** Relative mRNA concentrations of NF-κB p-65, p105/50 in SUM-159 luc cells. Data represent as the means ± S.D. of three independent experiments (n=3).

Five mg/kg of Britanin was injected into the tail veins of BALB/c mice in the low dose test group, 10 mg/kg of Britanin was injected into the mice in the high dose test group, and an equal volume of saline was injected into the tail veins of mice in the control group. Then, the tumor tissue was harvested for histopathological analysis after 72 h (**Figures 5A, D**). The TUNEL experiment results showed that Britanin treatment induced apoptosis in human breast cancer cells, and apoptosis was induced to a greater extent in the MDA-MB-231 cells than the SUM-159 cells (**Figures 5B–F**). The number of apoptotic cells tumors in athymic nu/nu mice treated with Britanin were significantly more than those in the mice of the placebo control group (P < 0.001). Furthermore, we used western blot and qRT-PCR to determine the effect of Britanin on the p-65, p105/p-50, and p-p65 in MDA-MB-231 luc cells and SUM-159 luc cells treated for 24 h. The p-50 protein expression concentrations decreased 24 h after the Britanin interventions when compared to non-treated group values (18.2% in MDA-MB-231 luc cells; 40.7% in SUM 159 luc cells, **Figures 6A, B**), although p-50 mRNA concentration levels increased 86.3% in MDA-MB-231 luc cells and 90.7% in SUM 159 luc cells by qRT-PCR analysis (**Figures 6C, D**). The p-p-50 protein expression levels decreased 24 h after the Britanin interventions when compared to non-treated group values (32.6% in MDA-MB-231 luc cells, 16.5% in SUM 159 luc cells) (**Figures 6A, B**). No significant differences in p-65 protein expression were observed after the Britanin intervention by western blot analyses (**Figures 6A, B**), although p-65 mRNA concentration levels increased 11.9% in MDA-MB-231 luc cells and 16.5% in SUM 159 luc cells by qRT-PCR analysis (**Figure 6C**).

### Molecular Docking for Binding Mode Prediction

**Figure 7:**
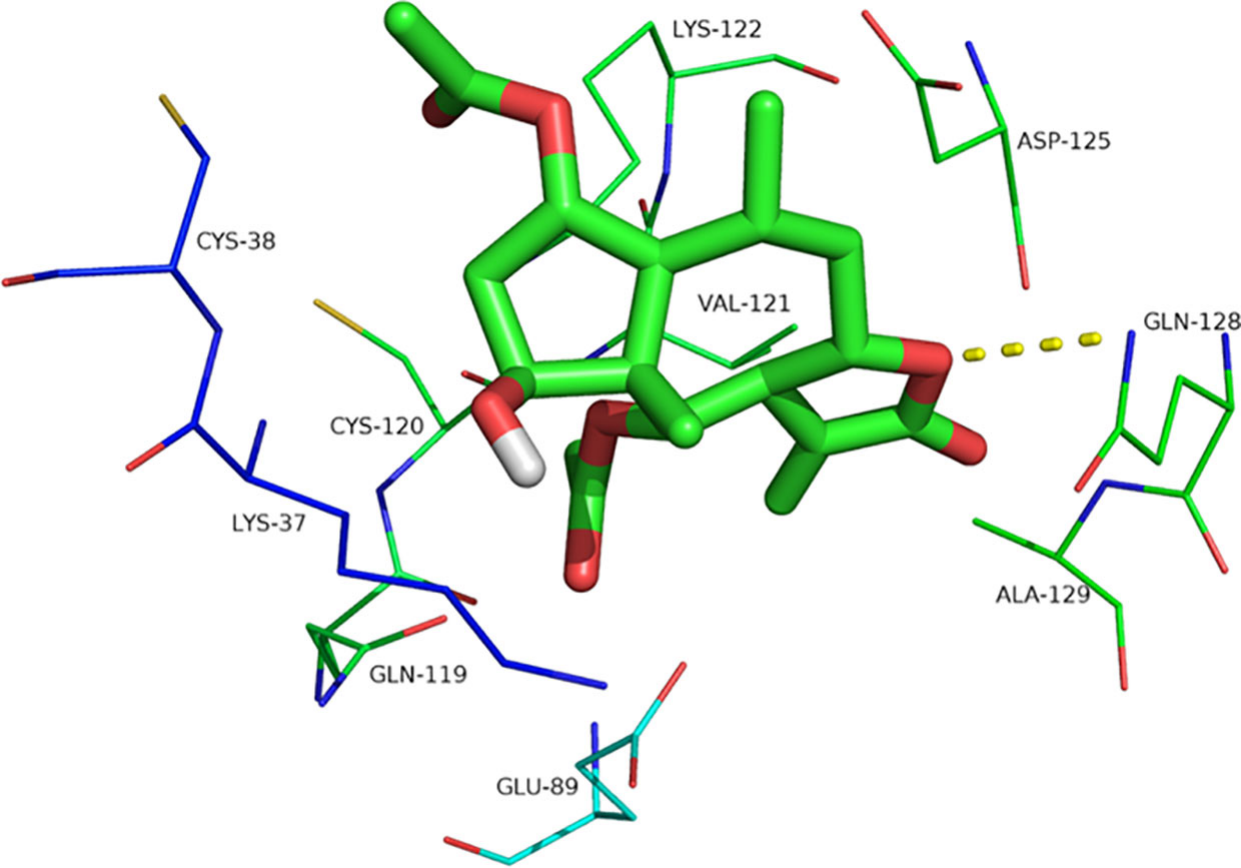
The interaction site of Britanin with p-65 by docking simulations. The p-65 is represented in the stick model and compound Britanin is drawn in green bold stick model.

Britanin docked into the binding pocket of p-65 (PDB coded: 1IKN), and the optimal energy conformation was selected by analyzing the results of 100 docking iterations. Ligands enter the target protein active sites mainly through hydrophobic and van der Waals chemical forces and interact with amino acid residues, including Lys37, Cys38, Gln89, Gln119, Cys120, Val121, Lys122, Asp125, Gln128, and Ala129. The docking energy is −6.07, and the binding activity of the ligand to the target protein is noncovalent. Oxygen atoms in the five-membered ring of the ligand form hydrogen bonds with Gln128, and the hydrogen bond length is 3.1 Å. The formation of hydrogen bonds is beneficial to small molecule-targeting proteins, thus inhibiting p-65 activity (**Figure 7**).

## Discussion

Triple-negative breast cancer (TNBC) is defined as a subtype of breast cancer and has the features of aggressive behavior, poor prognosis and no standard chemotherapy protocols. A comparison of TNBC with normal breast tissue showed substantial changes in the NF-κB signaling pathway that controls angiogenesis and tumorigenesis. Thus, NF-κB p-65 is a common and effective therapeutic drug target. Many plant-derived compounds inhibit the NF-κB pathway in breast cancer, such as triptolide parthenolide, hirsutine, and andrographolide. Britanin is a guaiacyl-type sesquiterpene lactone and an inhibitor of the NF-κB pathway that controls inflammatory responses. Therefore, we evaluated the anti-TNBC activity of Britanin using athymic nu/nu mice implanted with MDA-MB-231 luc and SUM-159 luc cells expressing a luciferase reporter gene *in vivo* by BLI and by measurement of tumor size *in vitro*. The results obtained from the BLI and measurement of the size of the tumors showed that Britanin inhibited the proliferation of tumor cells. Furthermore, the tumors in athymic nu/nu mice treated with Britanin were smaller than those in the mice treated with the placebo control. Thus, Britanin inhibited the proliferation of tumor cells effectively. Then, the mechanism of the anti-tumor activity of Britanin was studied by colony formation, Transwell migration. Britanin inhibited the proliferation of tumor cells. By western blot and qRT-PCR analysis, Britanin treatment inhibited the NF-κB pathway and reduced the metastatic and proliferative potential of TNBC cells. The anticancer mechanism was analyzed. The molecular docking results revealed that Britanin could covalently bind to the p-65 protein. These results of this study strongly support the investigation of Britanin as a promising new natural anticancer phytochemical for the treatment of TNBC refractory to currently available anticancer drugs.

In our study, the MDA-MB-231 cells derived from the pleural effusions of a Caucasian breast cancer patient and SUM-159 cells derived from a noninflammatory metaplastic patient selected for this screen were used to elucidate whether Britanin inhibits cell growth. In the *in vivo* BLI process, the luciferase report gene was transfected and used to label both of the above cell types. Cell lines stably expressing luciferase were established. The BLI *in vivo* results showed that there were no cell-specific effects by this method. The luminescence intensities of ROIs after treatment with Britanin were examined from the bioluminescence images continuously. And the trend in these results were similar with the trend in the tumor volume growth values. The experimental results show that there is a difference between tumor volume measured *in vivo* and tumor volume dissected. Because the tumor sizes of the experimental group and control group are different, their accurate measurement may be easily influenced by subjective factors. It is worth noting that by the traditional method the difference in tumor size between the test group and control group of mice was not evident on the 9th day and could not be observed until after the 14th day. However, from the first observation point (4th day or 9th day), the ROI value of the experimental group increased less than that of the control group. Meanwhile, real-time monitoring of BLI adds information to the effects of different Britanin concentrations and enables consecutive observation. Noninvasive examination *in vivo* combined with conventional and classical methods. The metabolically active cells in the BLI response, especially in small tumors, can be accurately detected, avoiding the impact of factors such as peritumoral edema for the traditional tumor volume measurement. Thus, Bioluminescence assays are more sensitive than traditional methods for detecting tumor size *in vivo* and *vitro*.

The TUNEL analysis revealed that compared to the control treatment, Britanin treatment markedly induced apoptosis in MDA-MB-231 and SUM-159 cells. The results herein indicate that Britanin markedly inhibits cell proliferation in MDA-MB-231 and SUM-159 human TNCB cancer cells in a dose- and time-dependent manner. The western blot and qRT-PCR analysis results showed that the expression of p-p65 of the test group cells was lower than that of the control group, and the total expression of p-65 has no difference between the test group and the control group. And NF-κB p-65 and p-50 mRNA levels had less than 10% increase. NF-κB is a dimeric form of transcription factor family ubiquitous in eukaryotes. In the cytoplasm, the NF-κB subunits p-65 and p-50 exist as heterodimers and form a complex with its inhibitory protein IκB. This complex in the cytoplasm covered the nuclear localization signal of the p-65, so the complex was in an inactive state. The p-65 was phosphorylated to exert its transcriptional effect. In addition, Britanin contains sesquiterpenoid lactones with α-methylene-γ-butyrolactone moiety. The binding mode prediction is that Michael-type thiols bind to cysteine sulfhydryl groups of p-65.

In conclusion, the results of the present study show a novel use of Britanin in mitigating human TNBC proliferation and inducing apoptosis *via* the inhibition of the NF-κB pathways. Another conclusion is that this bioluminescence evaluation method allows the simultaneous visualization of changes in tumor volume and could be applied to new types of drug screening, discovery, and development.

## Data Availability Statement

The raw data supporting the conclusions of this article will be made available by the authors, without undue reservation, to any qualified researcher.

## Ethics Statement

All animal studies carried out in compliance with the Guide for the Care and Use of Laboratory Animal Resources and approved by the University of Xi'an Jiaotong Animal Care and Use Committee (number XY-AUC-2017-213).

## Author Contributions

XX carried out biological experiments. YG carried out animal experiments. GD and HL analyzed data and review the manuscript. LW and DC wrote the paper and led the research.

## Funding

This work was supported, in part, by the National Key R&D Program of China under Grant No. 2018YFC0910600 and the Natural Science Basic Research Plan in Shaanxi Province of China under Grant No. 2019JQ-519 and 2018JM7072.

## Conflict of Interest

The authors declare that the research was conducted in the absence of any commercial or financial relationships that could be construed as a potential conflict of interest.
