# Supplementary figures and images for: Bioluminescence Imaging-Based Assessment of the Anti-Triple-Negative Breast Cancer and NF-Kappa B Pathway Inhibition Activity of Britanin
Source: Front Pharmacol. 2020 May 5;11:575. doi: 10.3389/fphar.2020.00575 (PMC7215071; doi:10.3389/fphar.2020.00575)

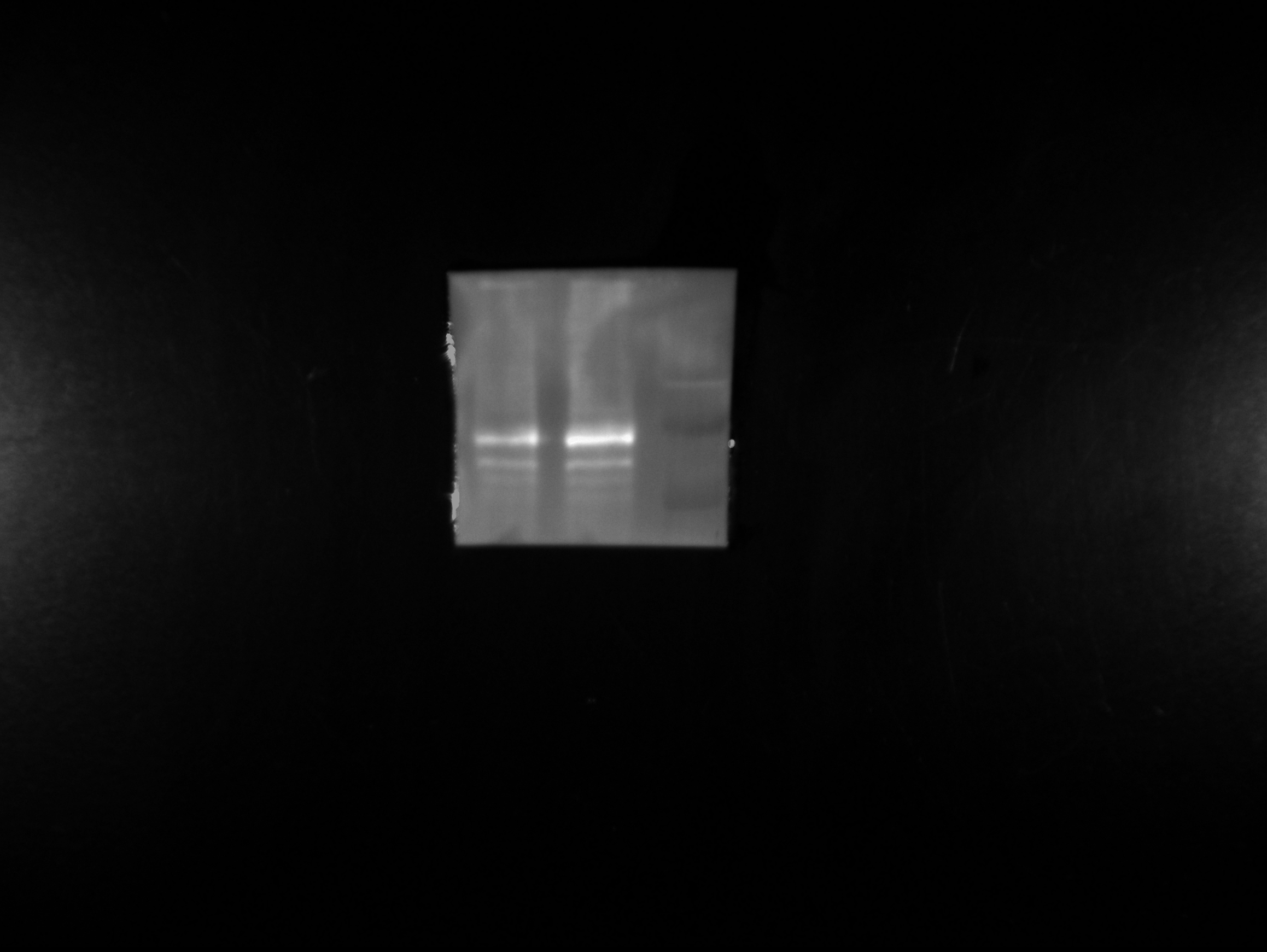

Supplement: Supplementary file 1 [file DataSheet_1.zip › Data Sheet 1/1.tif]

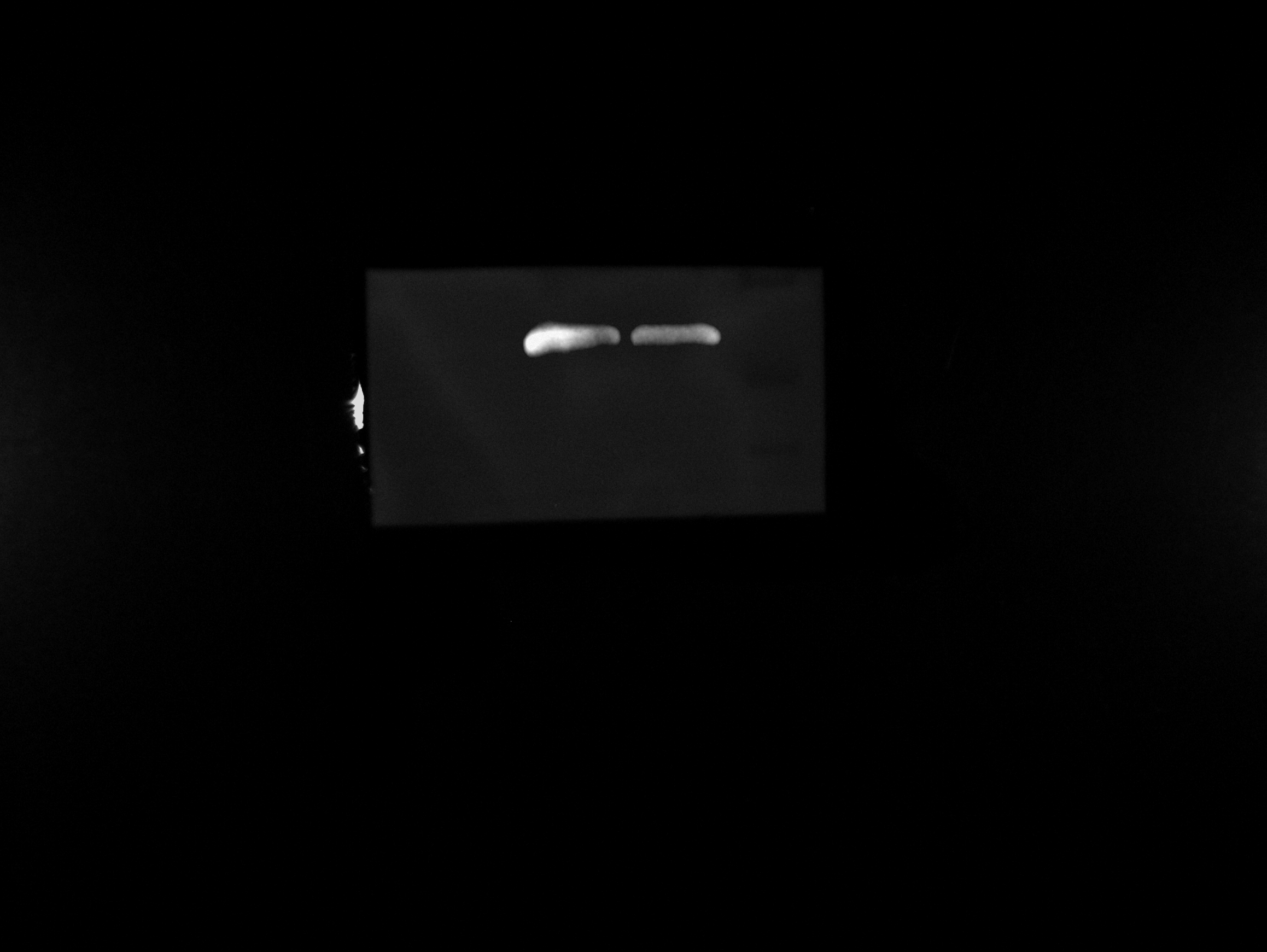

Supplement: Supplementary file 1 [file DataSheet_1.zip › Data Sheet 1/10.tif]

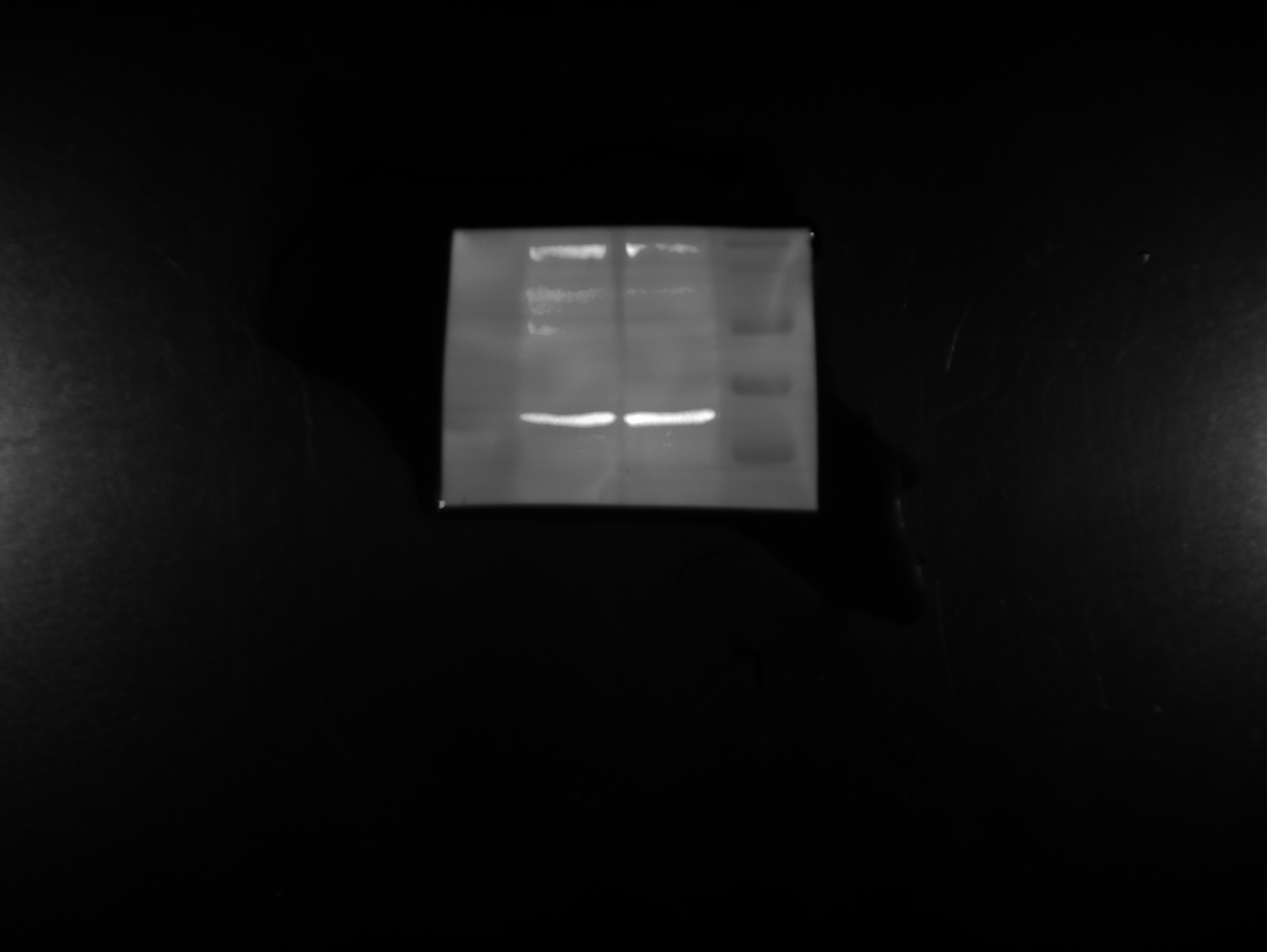

Supplement: Supplementary file 1 [file DataSheet_1.zip › Data Sheet 1/11.tif]

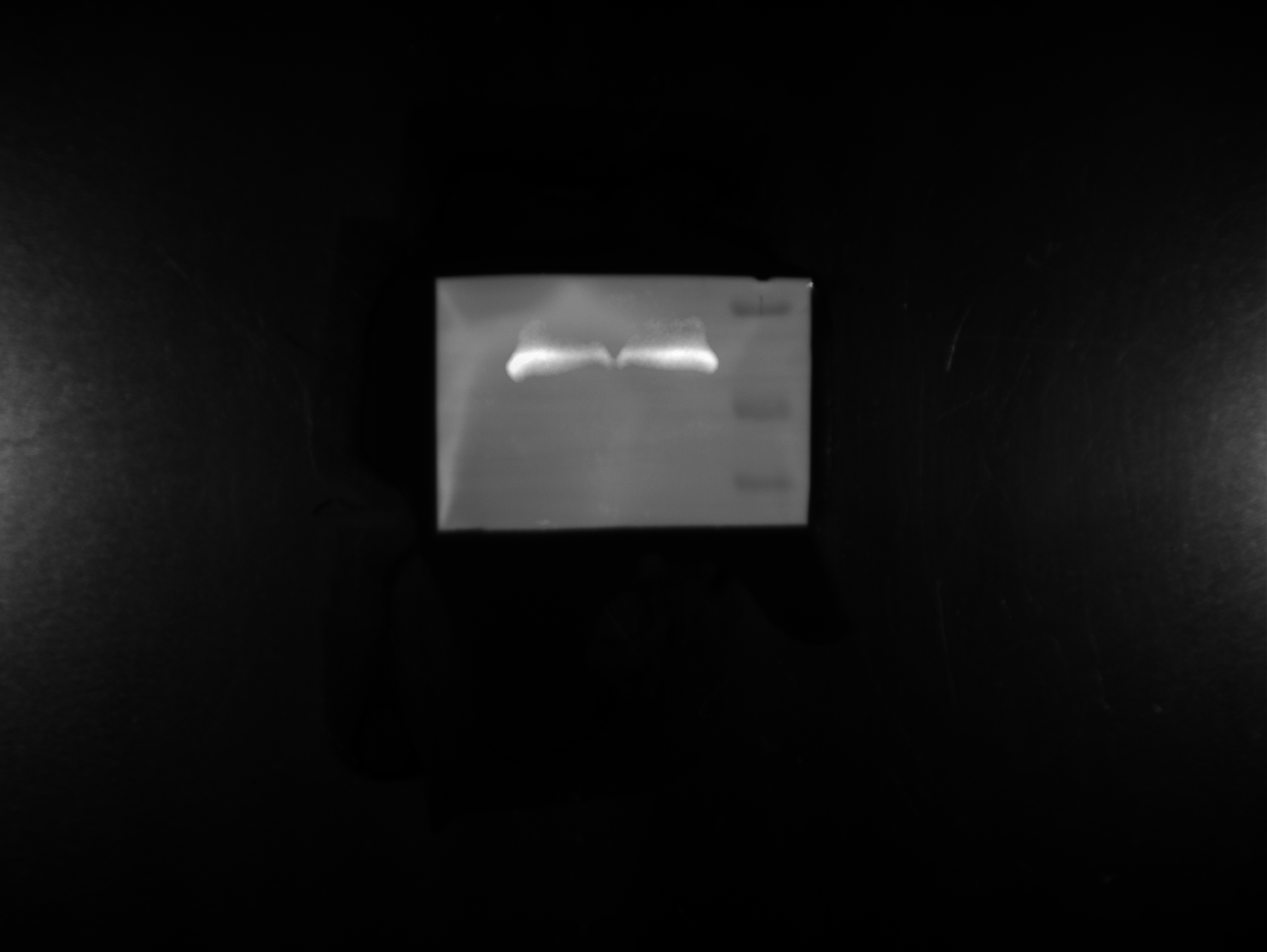

Supplement: Supplementary file 1 [file DataSheet_1.zip › Data Sheet 1/12.tif]

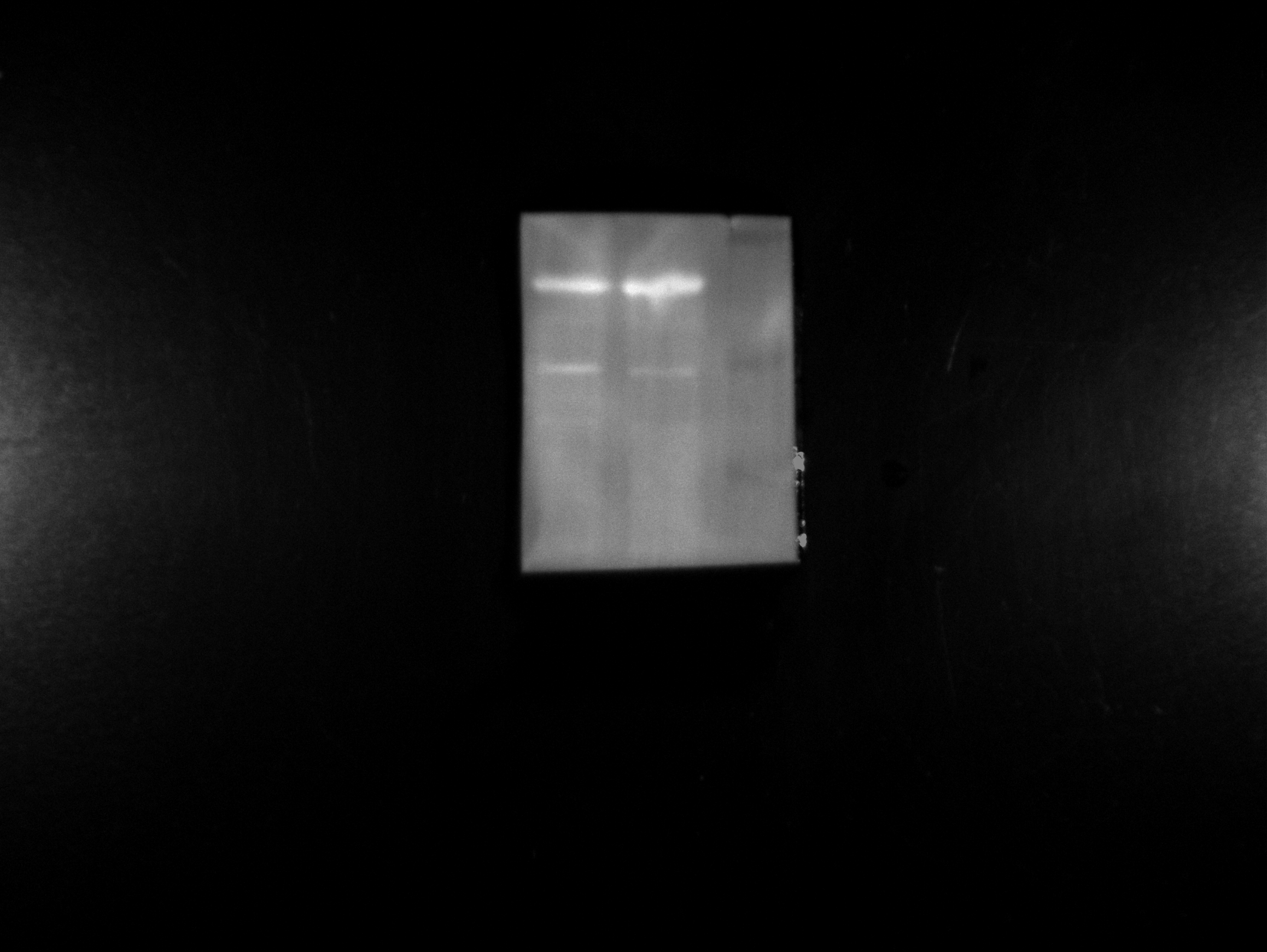

Supplement: Supplementary file 1 [file DataSheet_1.zip › Data Sheet 1/2.tif]

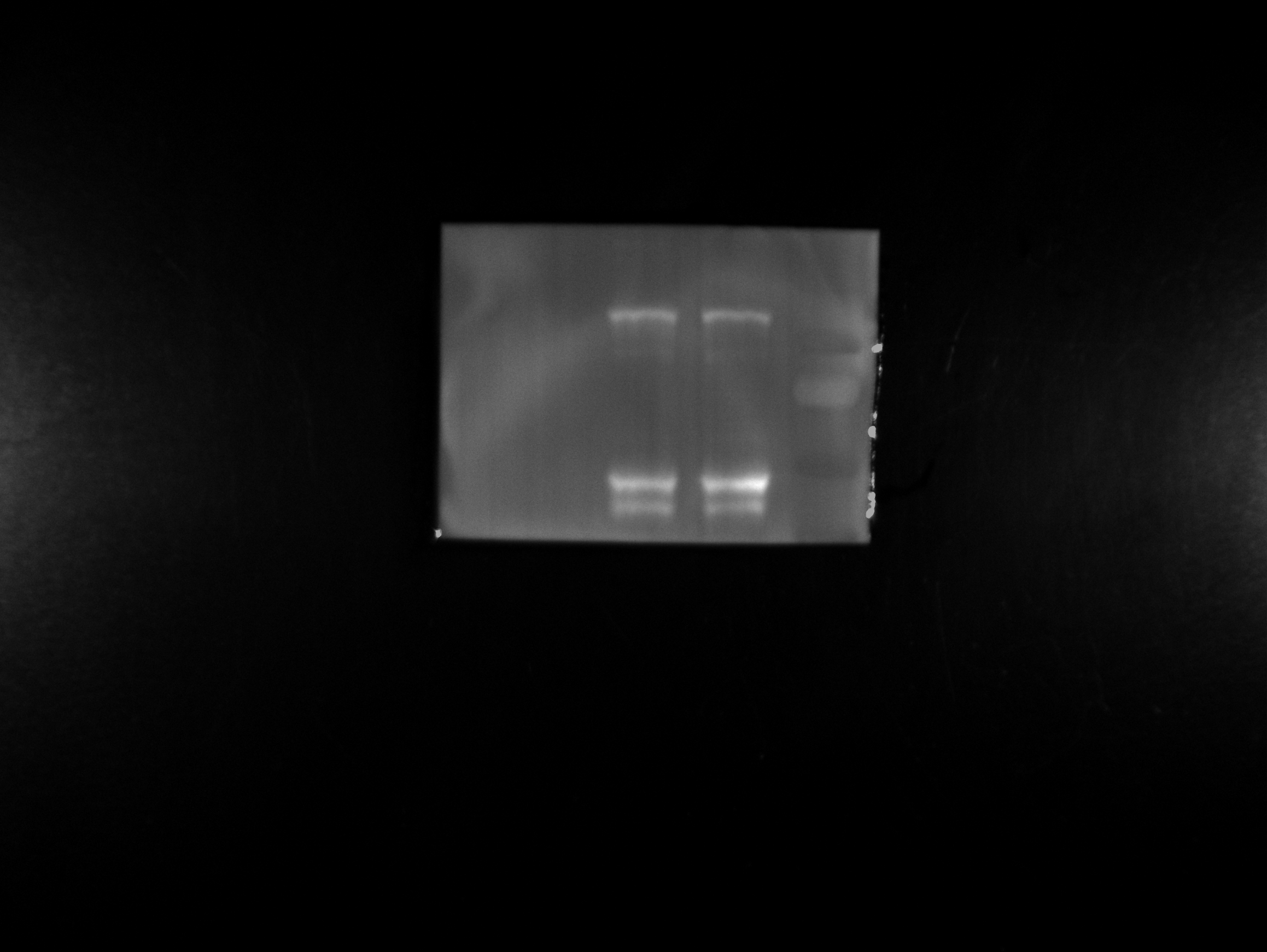

Supplement: Supplementary file 1 [file DataSheet_1.zip › Data Sheet 1/3.tif]

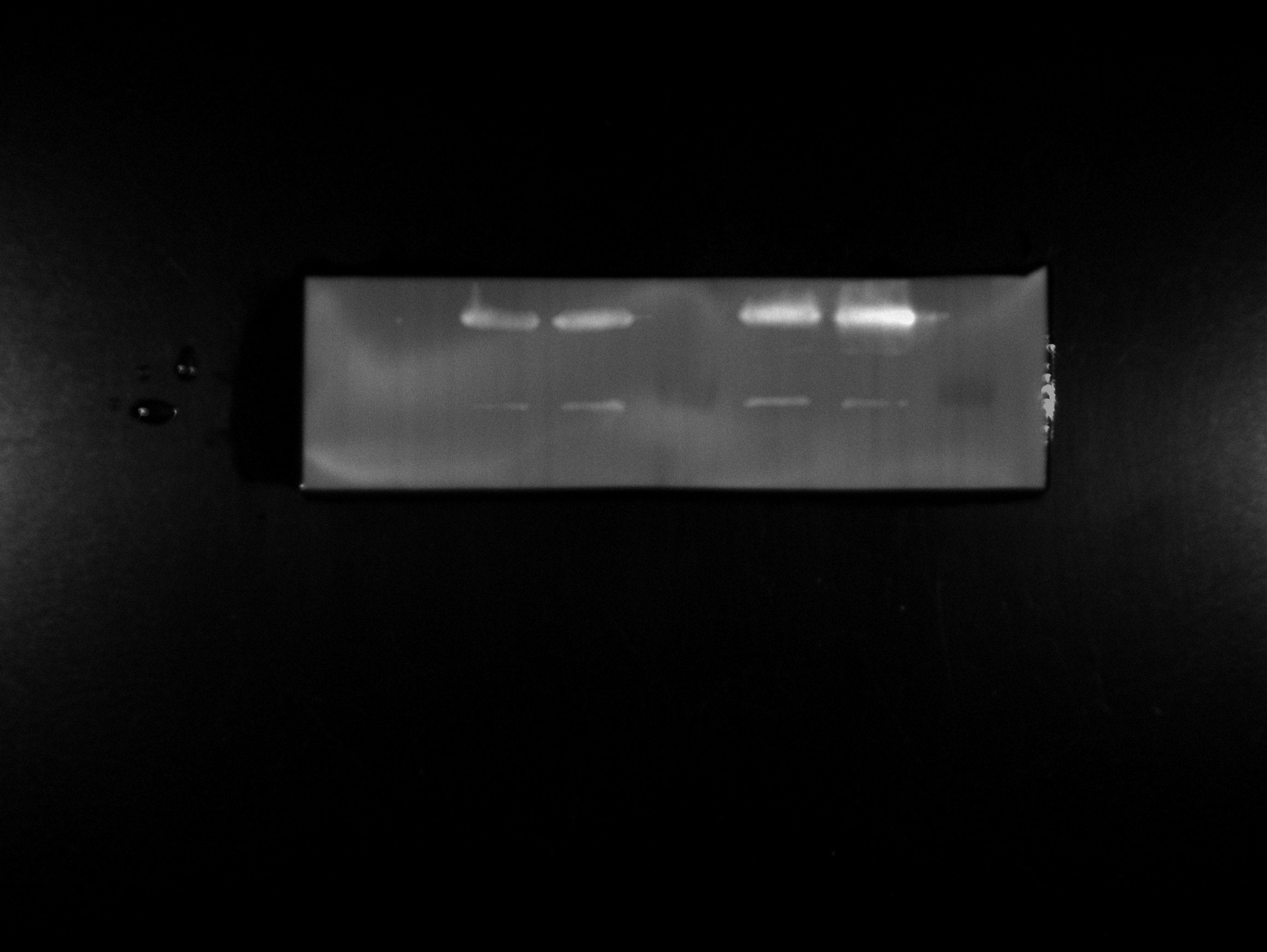

Supplement: Supplementary file 1 [file DataSheet_1.zip › Data Sheet 1/4.tif]

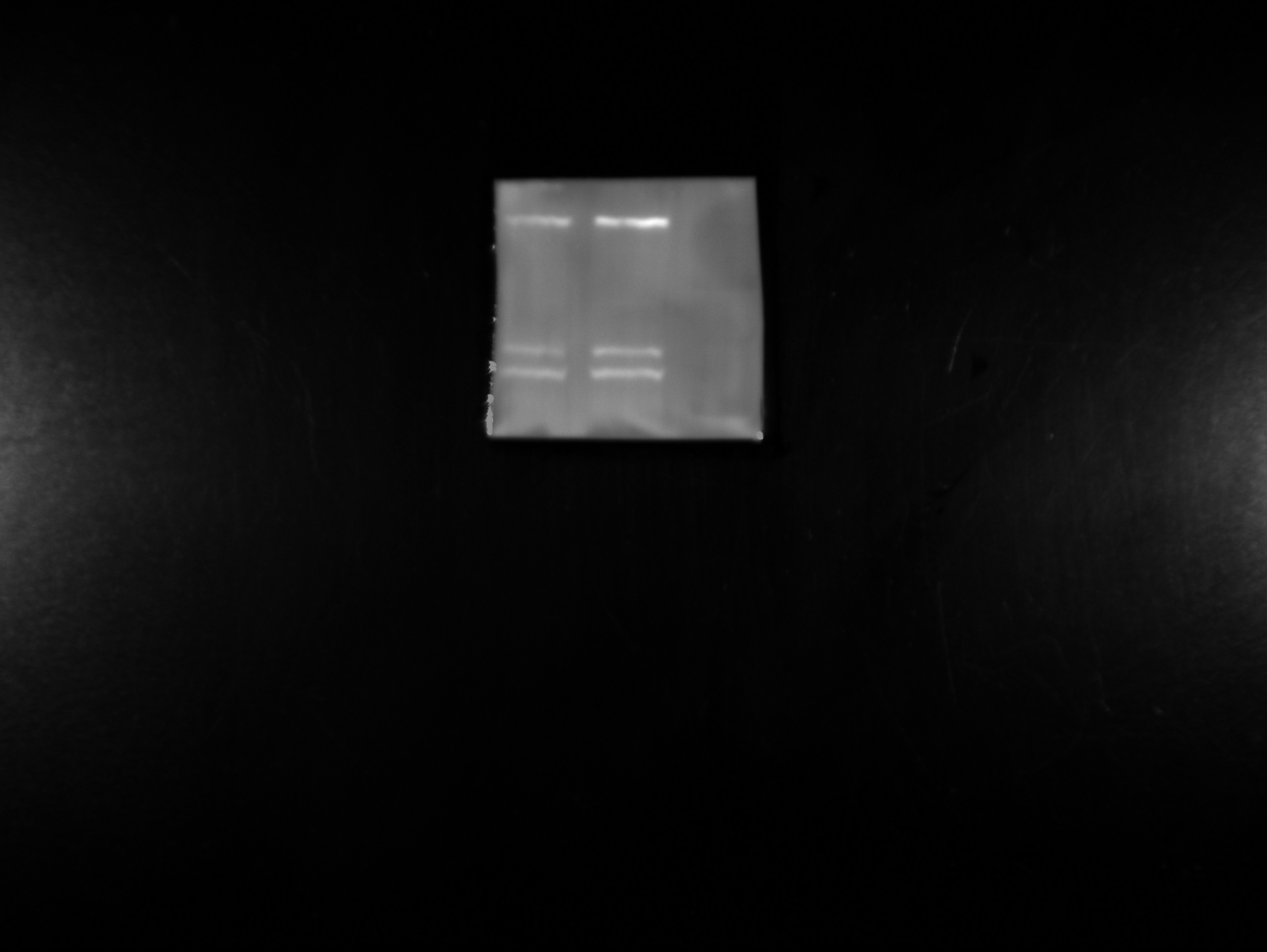

Supplement: Supplementary file 1 [file DataSheet_1.zip › Data Sheet 1/5.tif]

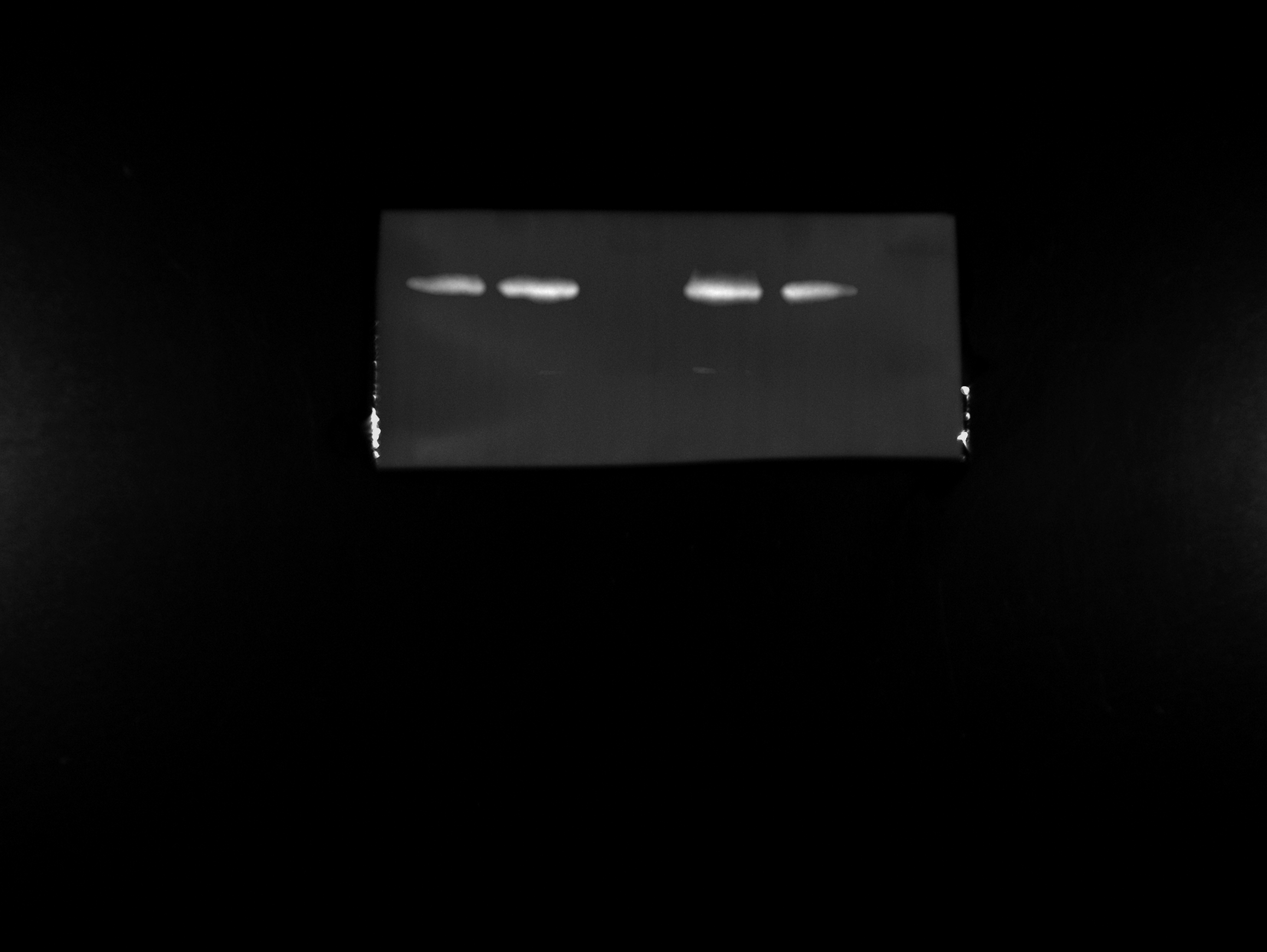

Supplement: Supplementary file 1 [file DataSheet_1.zip › Data Sheet 1/6.tif]

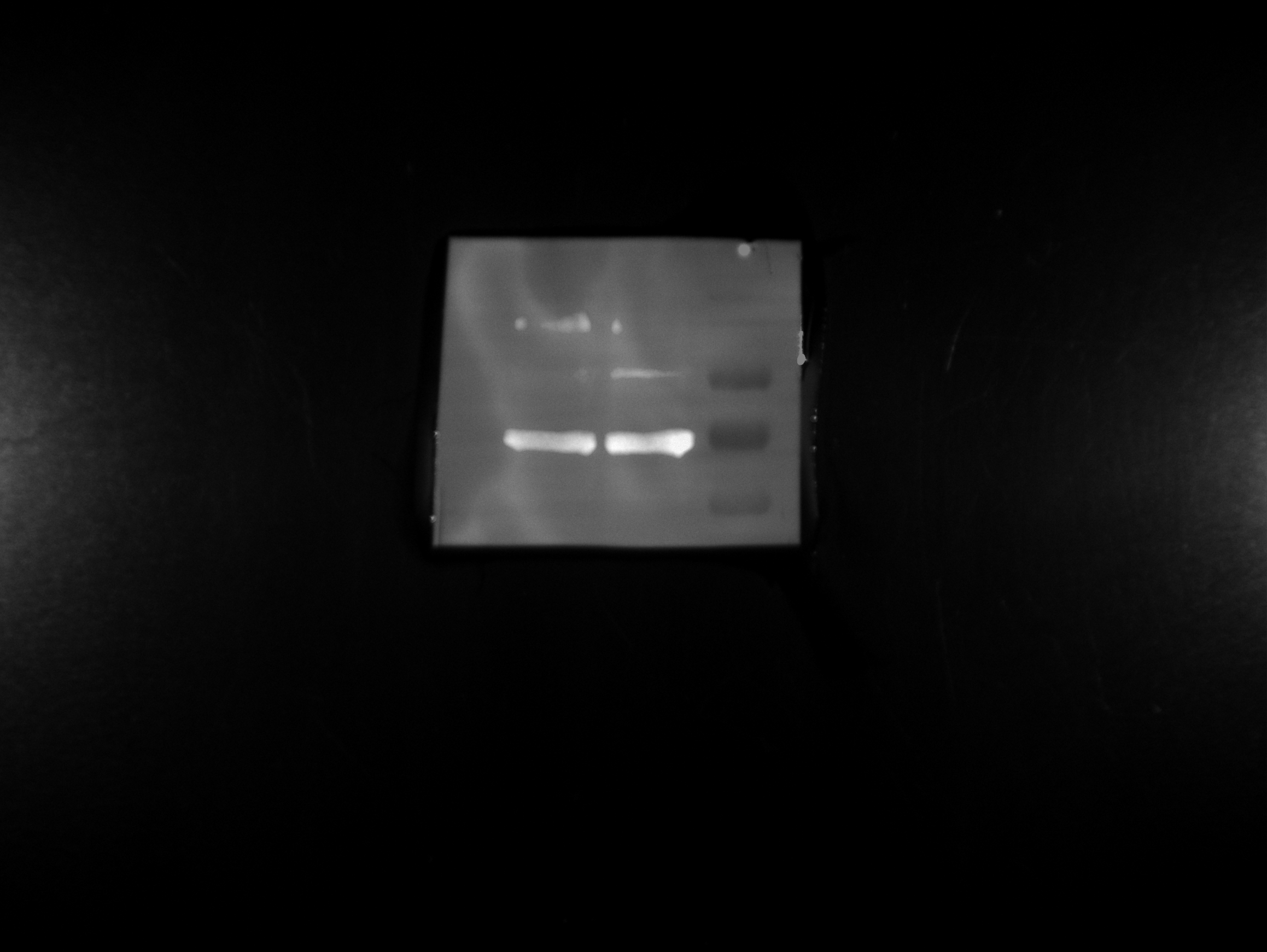

Supplement: Supplementary file 1 [file DataSheet_1.zip › Data Sheet 1/7.tif]

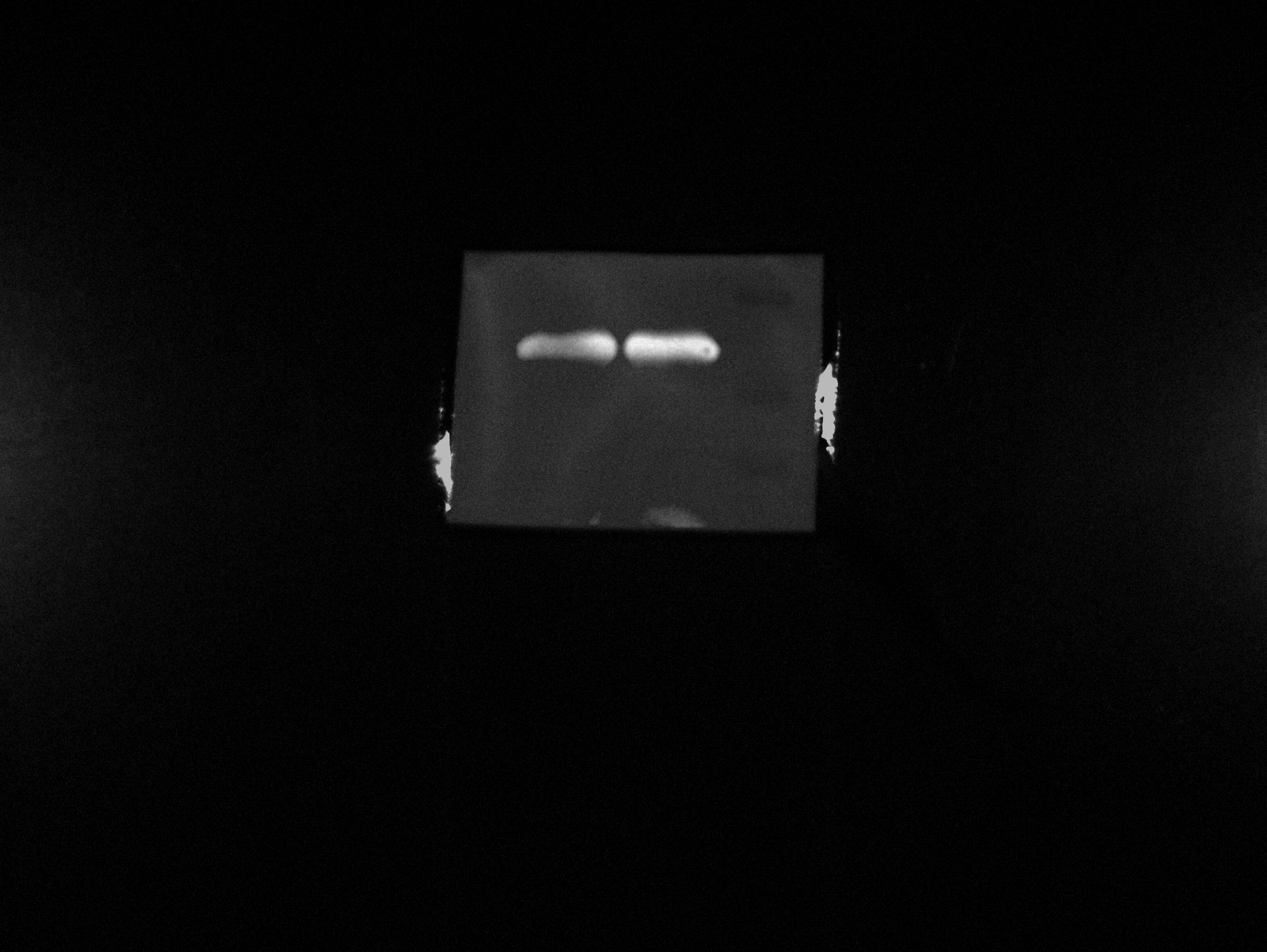

Supplement: Supplementary file 1 [file DataSheet_1.zip › Data Sheet 1/8.tif]

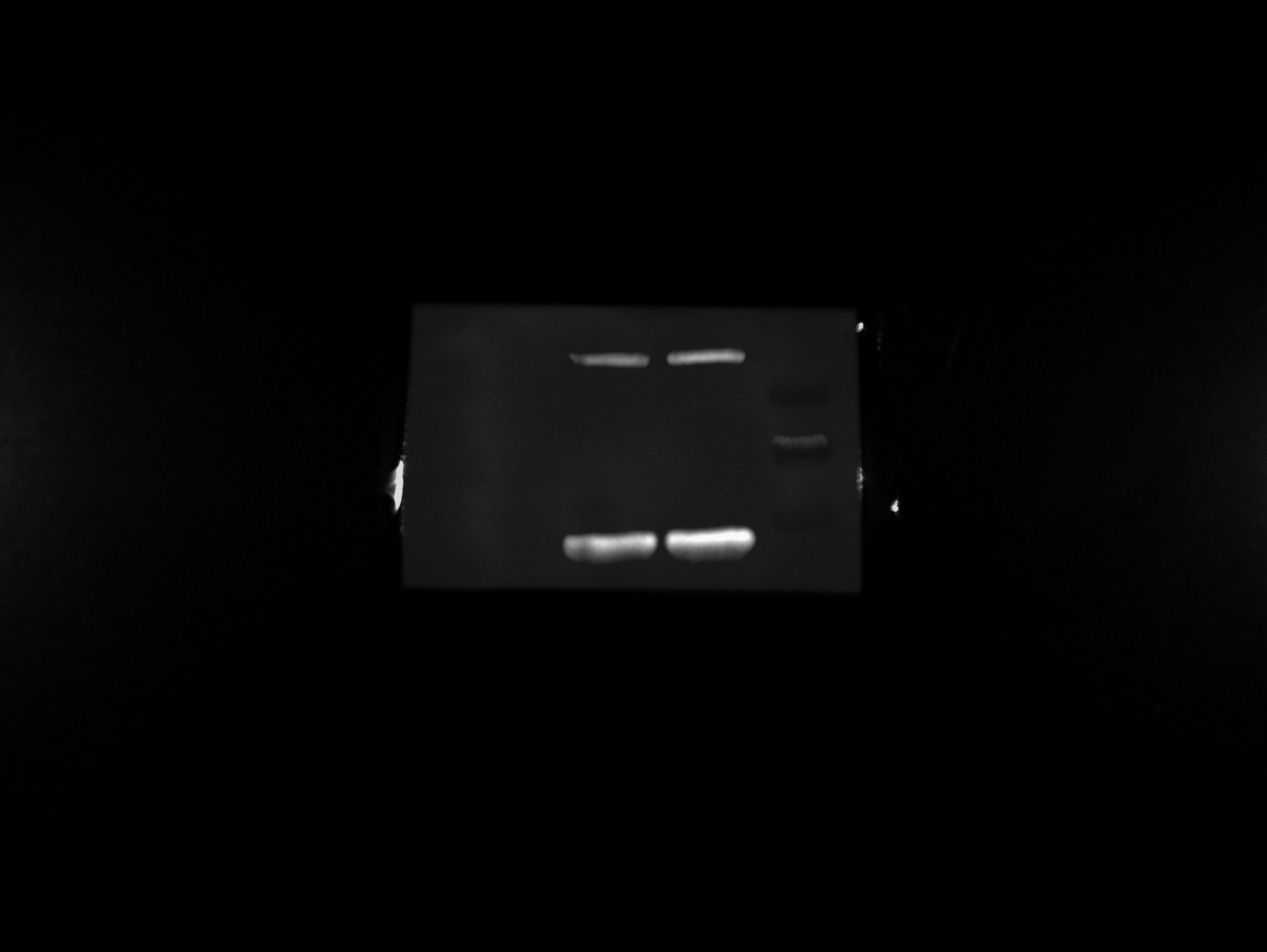

Supplement: Supplementary file 1 [file DataSheet_1.zip › Data Sheet 1/9.tif]
