# Supplementary material for: Bioluminescence Imaging-Based Assessment of the Anti-Triple-Negative Breast Cancer and NF-Kappa B Pathway Inhibition Activity of Britanin
Source: Front Pharmacol. 2020 May 5;11:575. doi: 10.3389/fphar.2020.00575 (PMC7215071; doi:10.3389/fphar.2020.00575)
Supplement: Supplementary file 2 [file DataSheet_2.pdf]

Bioluminescence imaging-based assessment of the anti-triple-negative breast cancer and NF-kappa B pathway inhibition activity of Britanin

Xinyi Xu[1,#], Yingying Guo[1,#], Getao Du[1], Huifang Liu[1], Lin Wang[2,*], Dan Chen[1,*]

[1] Engineering Research Center of Molecular and Neuroimaging, Ministry of Education, School of Life Science and Technology, Xidian University, Xi'an, Shaanxi, P. R. China
[2] School of Information Sciences and Technology, Northwest University, Xi'an Shaanxi, P. R. China
*** Correspondence:**
Dan Chen: dchen@xidian.edu.cn
Lin Wang: wanglinmig@gmail.com

Supplementary Material

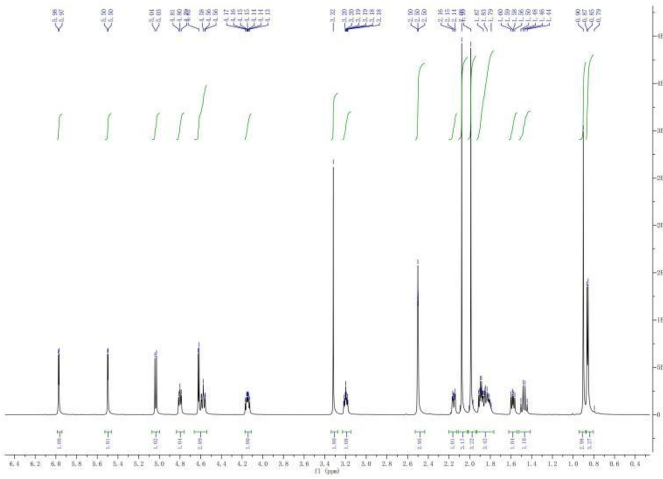

Supplementary Figure S1. The nuclear magnetic resonance spectroscopy of Britanin at 600 MHz. ^{1}H NMR (600 MHz, DMSO-d6, δ, ppm, J/Hz): 0.79 (3H, d, J = 6.0,), 0.87 (3H, s), 1.46 (2H, m), 1.58 (2H, m), 1.85 (1H, m), 1.99 (1H, m), 2.08 (3H, s), 2.15 (3H, s), 2.50 (2H, m), 3.20 (2H, m), 3.32 (1H, m), 4.15 (1H, dd, *J* = 11.0, 9.0), 4.59 (1H, m), 4.80 (1H, t, *J* = 5.0), 5.03 (1H, d, *J* = 12.0), 5.50 (2H, d, *J* = 3.0), 5.97 (2H, d, *J* = 3.0).

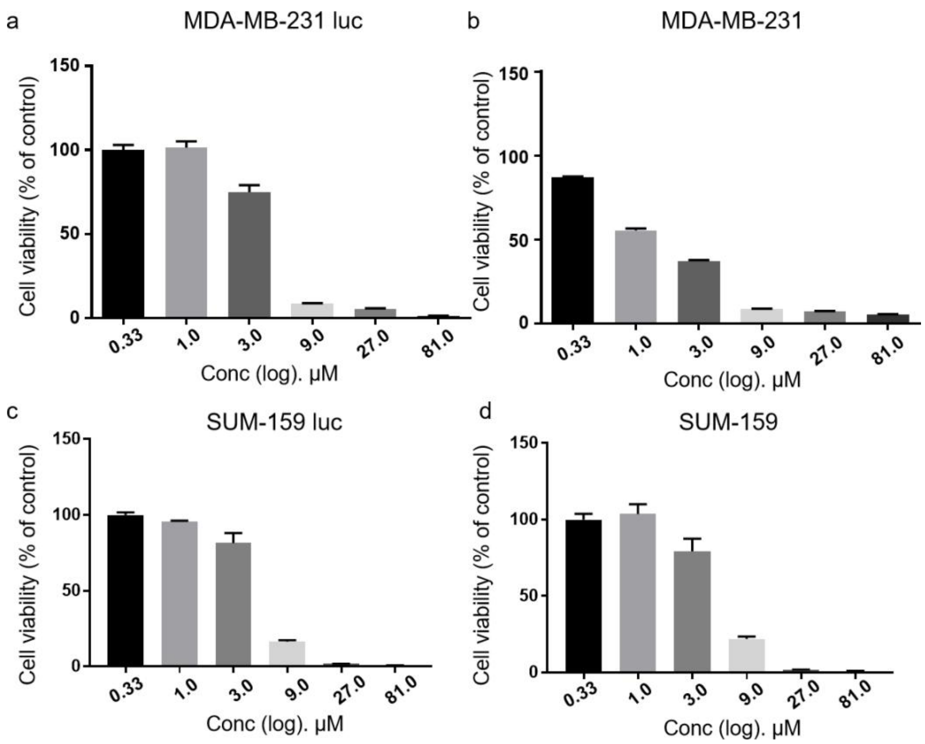

Supplementary Figure S2. The effect of britanin on the survival rate of MDA-MB-231 luc cells (a), MDA-MB-231 cells (b), SUM-159 luc cells (c) and SUM-159 cells (d) were quantified by MTT method. Britanin was administered at concentrations of 0.33, 1.0, 3.0, 9.0, 27.0 and 81 μM for 72 h. The results are presented as the mean ± SD of three independent experiments; the SD is denoted by error bars.

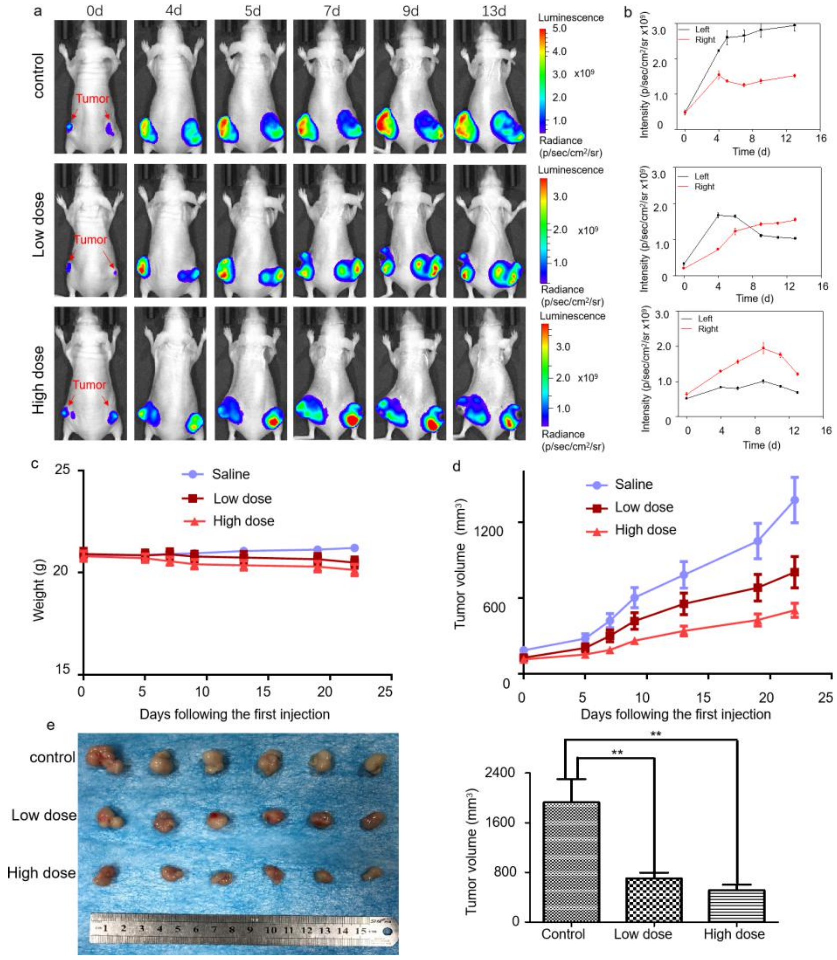

Supplementary Figure S3. The activity evaluation of Britanin on breast cancer model mouse *in vivo*. (a) Representative bioluminescence images recorded before and after Britanin injections of low dose (5 mg/kg) and high dose (10 mg/kg) by intraperitoneal injection in SUM-159 luc cells inoculated mice tumor models compared with an untreated control group (day 4, day 5, day 7 day 9 and day 13). (b) Quantification of bioluminescence intensity by ROIs that encompass the tumor. Data represent as the means ± S.D. (c) The mouse was weighted before and after Britanin injections (day 5, day 7, day 9, day 13, day 19 and day 22). (d) Tumor volume was calculated before and after Britanin injections (day 5, day 7, day 9, day 13, day 19 and day 22). (e) Photograph of excised tumors from the control, low dose test treatment group and high test treatment group. Graph represents the average weight of the tumor. $^{**}p < 0.01$.
