# Supplementary material for: Bioluminescence Imaging-Based Assessment of the Anti-Triple-Negative Breast Cancer and NF-Kappa B Pathway Inhibition Activity of Britanin
Source: Front Pharmacol. 2020 May 5;11:575. doi: 10.3389/fphar.2020.00575 (PMC7215071; doi:10.3389/fphar.2020.00575)
Supplement: Supplementary file 1 [file DataSheet_1.zip › Data Sheet 1/the caption of Data Sheet 1.doc]

1.Western blots of proteins extracted from MDA-MB-231 luc cells treated with or without Britanin with the NF-κB p-65 antibodies.

2. As the 1 control, western blots of proteins extracted from MDA-MB-231 luc cells treated with or without Britanin with the GAPDH antibodies

3.Western blots of proteins extracted from MDA-MB-231 luc cells treated with or without Britanin with the p105/p50 antibodies.

4.As the 3 control, western blots of proteins extracted from MDA-MB-231 luc cells treated with or without Britanin with the GAPDH antibodies.

5.Western blots of proteins extracted from MDA-MB-231 luc cells treated with or without Britanin with the p-p65 antibodies.

6.As the 5 control, western blots of proteins extracted from MDA-MB-231 luc cells treated with or without Britanin with the GAPDH antibodies.

7.Western blots of proteins extracted from SUM-159 luc cells treated with or without Britanin with the NF-κB p-65 antibodies.

8. As the 7 control, western blots of proteins extracted from SUM-159 luc cells treated with or without Britanin with the GAPDH antibodies

9.Western blots of proteins extracted from SUM-159 luc cells treated with or without Britanin with the p105/p50 antibodies.

10.As the 9 control, western blots of proteins extracted from SUM-159 luc cells treated with or without Britanin with the GAPDH antibodies.

11.Western blots of proteins extracted from SUM-159 luc cells treated with or without Britanin with the p-p65 antibodies.

12.As the 11 control, western blots of proteins extracted from SUM-159 luc cells treated with or without Britanin with the GAPDH antibodies.
